# Growing Up in Times of COVID-19: When a Window of Opportunity is Temporarily Closed

**DOI:** 10.1007/978-3-030-65355-2_17

**Published:** 2021-03-20

**Authors:** Loes Keijsers, Anne Bülow

**Affiliations:** 1 Tilburg University, Tilburg, The Netherlands; 2 Tilburg University, Tilburg, The Netherlands; 3 Tilburg University, Tilburg, The Netherlands; 4 Tilburg University, Tilburg, The Netherlands; 5 Department of Developmental Psychology, Tilburg School of Social and Behavioral Sciences, Tilburg, The Netherlands; 6 Department of Psychology, Education & Child Studies, Erasmus School of Social and Behavioural Sciences, Rotterdam, The Netherlands

## Abstract

During the COVID-19 crisis, the governmental restrictions seriously affected the daily lives of adolescents (aged 12–25). They could not attend school, had to limit face-to-face contact with peers, and had to stay at home with their parents. This chapter combines insights from theoretical models on adolescent development with some of the first empirical findings of the impact of COVID-19 on adolescents. We will discuss how lockdown and social distancing measures affect mental health and well-being in a formative and vulnerable period in life. Specifically, the authors focus on delayed attainment of developmental tasks toward adulthood, the importance of friendships, and how parents can promote developmental growth and resilience in a “new common.” Advice is included on how future society can and should be shaped around the developmental needs, risks, and opportunities that characterize adolescence.

In the spring of 2020, COVID-19 spread across the globe and many governments took restrictive measures to prevent a further spread. Even though adolescents, here defined as youths aged 12–25 years, are not a high-risk group from an epidemiological viewpoint, school closures and social distancing measures had a tremendous impact on their daily lives. Adolescence is characterized by opportunities for personal growth, for establishing friendships that last a lifetime, for falling in love for the very first time, and for learning how to cope with stress. Simultaneously, adolescents are vulnerable for psychological problems, with approximately one out of five facing emotional problems, such as anxiety problems or depression (Kessler et al. 2005). In terms of social health, loneliness affects adolescents more than any other age group (Qualter et al. 2015). This chapter reflects on the psychological and social consequences of COVID-19 on adolescents while we are in the midst of the first wave. The aim is to provide concrete advice on how society, parents, and professionals can create optimal circumstances for promoting the growth of the next generations in a new common.

## Attainment of Developmental Tasks

When the body is ripe, and society requires, and the self is ready to achieve a certain task, the teachable moment has come. (Havighurst 1948)

Adolescents mature by the accomplishment of developmental tasks, by societies’ predefined stepping stones (Havighurst 1948). These tasks include forming friendships and romantic relationships, achieving independence from parents, rituals of entry into adulthood (e.g., graduation), and selecting and preparing for an occupation. Society as a whole and also parents and adolescents themselves have a timetable of expectations regarding when a child should be able to accomplish these tasks (Dekovic et al. 1997). For instance, 15 year olds should be able to maintain friendships and solve a conflict on their own. Being “on schedule” provides youths with a sense of pride and meaning in life and is related to emotional well-being. Delay on the other hand, comes with a price of increased psychological problems and lower self-esteem (Seiffge-Krenke and Gelhaar 2008). Hence, even in times of a crisis, society, educators, and parents need to allow for these critical maturation tasks to be attained at the right moment.

## Social Deprivation

As any other human being, adolescents have a fundamental need to belong, that is, to form and maintain high-quality, intimate, and stable relationships with others (Baumeister and Leary 1995). Whereas adults typically rely on their romantic partners for comfort, intimacy, and understanding, close friends play this pivotal role in the lives of teens (De Goede et al. 2009). In the context of close friendships, adolescents develop a set of social skills needed to function later in life, such as keeping promises, solving conflicts, and disclosing secrets (Frijns et al. 2013). Through the exploration of sexuality and romance with age-mates adolescents, they gradually learn how to engage in intimate relationships that are pleasant and long-lasting (van de Bongardt 2019). Moreover, in the ongoing search for the self, conversations with friends help an adolescent to form a stable identity (Reis and Youniss 2004). Friendships, in sum, are not only a source of great joy in adolescents’ lives, they are fundamental for learning how to cope with stress, how to maintain relationships, and form the basis for future growth and maturation.

During the COVID-19 outbreak in 2020, we assessed how the lockdown had affected friendships as part of the ADAPT project (Keijsers et al. 2017). Among 178 Dutch middle adolescents (age = 14.25 (SD = 1.63), 31% boys), we observed strong declines in time spent with friends, from 8 hours of face-to-face contact during weekdays before the lockdown to 2 hours after and, during weekend days, from 6.5 to 2 hours. Adolescents rapidly adapted to the new situation, as online contact with friends increased from 3 hours/day to 5.5 hours. Although this online communication may compensate for some of the negative effects of the sharp decrease in face-to-face contacts (Orben et al. 2020), it is still likely that adolescents missed important opportunities to obtain support and comfort from friends and romantic partners, in circumstances when these social resources were needed most. Social deprivation may also have affected their well-being. A rapid systematic review highlighted that 30–50% of the adolescents aged 12–24 were lonely during the COVID-19 lockdown (Loades et al. 2020). Feelings of loneliness may increase the risk of developing mental health problems especially when they last longer (Qualter et al. 2015; van Roekel et al. 2013). For this reason, scholars have warned of a steep increase in mental health problems in the upcoming period, including anxiety and depression (Golberstein et al. 2020; Loades et al. 2020).

## Independence from Parents

Parents play a pivotal role in helping adolescents to become adults who are resilient to stress and who function adaptively in society. During adolescence, families are challenged by the increasing developmental need of adolescents to decide things for themselves. Conflicts can easily emerge on topics such as tidying the bedroom, spending time on social media, or adhering to curfews. These negative interactions are in fact helpful in pushing the parent–child relationship from a hierarchically structured relationship in which the parent has the final say to a more horizontally structured relationship with the more democratic decision-making (Branje et al. 2011). Hence, developmental growth and maturation take place when parents release control and trust their children to make wise decisions on their own (Keijsers and Poulin 2013).

In the ADAPT project (Keijsers et al. 2017), we observed the opposite change pattern. During the lockdown, instead of releasing control, parents became more protective and controlling. New rules were established by parents, mostly to reinforce governmental rules of hygiene and social distancing, such as not being allowed to see friends. Parents also introduced rules to structure the lives of their children, such as doing homework and getting up in time. Longitudinal analyses of eight repeated assessments indeed revealed a significant decrease in autonomy-supportive parenting during the lockdown (Bülow et al. 2020). As prohibition of contacts with friends and restriction of autonomy directly undermine the opportunities for growth, we anticipated a rise in conflicts and opposition (Van Petegem et al. 2017). However, this was only found for some families. Apparently, most parents manage to introduce and explain rules in a democratic way, and children find most of the novel rules legitimate. In fact, in some families, the increase in time spent together may have created opportunities to reinforce the relationship and improve communication (Keijsers et al. 2010). On the other hand, stress due to health or financial concerns may also trigger the use of a repressive parenting strategy, including guilt induction and love withdrawal (Van Der Kaap-Deeder et al. 2019). Such psychological control may impede on maturation processes and lead to internalizing problems, such as depressive feelings or anxiety, among adolescents. C’est la ton qui fait la musique when it comes to finding the balance between protection and promoting independence.

## Promoting Developmental Growth in the New Common

Adolescents are in the midst of establishing an important foundation for developmental growth and future well-being. Adolescents need friendships, independence from parents, and rituals that mark the entry to adulthood and new phases in life, such as graduation ceremonies. The COVID-19 situation has strongly affected each of these domains. One can never truly predict how an individual’s life course is affected by such a temporary situational change. In general, most adolescents will probably be resilient, and negative experiences and a short-term reduction in well-being do not doom them to an adult life full of ill-being and psychosocial problems (Cicchetti and Rogosch 2002). Likewise, adolescents who are resilient during this stressful period cannot comfortable rely on a problem-free future. The longer-term impact can only be derived from rigorous future scientific studies on adolescents. However, this should not prevent society from creating optimal circumstances for growth while we are still in the midst of the crisis.

First of all, as they are vulnerable to psychological problems, it is of pivotal importance to monitor and support adolescents. The current COVID-19 situation is one of many changes for adolescents. Social distancing undermines social support of their most intimate companions, which may lead to loneliness and decreased well-being. Accessible tools and informal and low-key professional guidance are needed to prevent psychosocial problems, such as depression (Golberstein et al. 2020). Examples include eHealth tools that challenge adolescents to cope with stress, such as our recently released Grow It! application (Hillegers et al. 2020).

Second, face-to-face contact with friends is not a luxury for adolescents; it is a developmental need. Social media is a blessing for adolescents, but it cannot compensate for the richness of learning experiences and support that face-to-face interactions provide. Third, during the lockdown measures, adolescents’ potential for growth and resilience was hindered because parents had to restrict opportunities for independent decision-making. In sum, in the new common, parents, teachers, and professionals should protect adolescents only when needed and allow for autonomy, independent decision-making, and contact with their friends whenever possible. Even when society is locked down, the window of opportunity for growth needs to be open for adolescents. After all, if adults support youngster in acquiring social skills and personal resources during a formative period in life, the next generation will be better able to cope with stress and societal changes to come.
